# Deep sequencing analysis of the developing mouse brain reveals a novel microRNA

**DOI:** 10.1186/1471-2164-12-176

**Published:** 2011-04-05

**Authors:** King-Hwa Ling, Peter J Brautigan, Christopher N Hahn, Tasman Daish, John R Rayner, Pike-See Cheah, Joy M Raison, Sandra Piltz, Jeffrey R Mann, Deidre M Mattiske, Paul Q Thomas, David L Adelson, Hamish S Scott

**Affiliations:** 1 Department of Molecular Pathology, SA Pathology and Centre for Cancer Biology, P.O. Box 14 Rundle Mall Post Office, Adelaide, SA 5000, Australia; 2 School of Medicine, Faculty of Health Sciences, University of Adelaide, Adelaide, SA 5005, Australia; 3 Department of Obstetrics and Gynaecology, Faculty of Medicine and Health Sciences, Universiti Putra Malaysia, 43400 UPM Serdang, Selangor DE, Malaysia; 4 School of Molecular and Biomedical Science, Faculty of Sciences, University of Adelaide, Adelaide, SA 5005, Australia; 5 Department of Human Anatomy, Faculty of Medicine and Health Sciences, Universiti Putra Malaysia, 43400 UPM Serdang, Selangor DE, Malaysia; 6 eResearchSA, University of Adelaide, North Terrace, Adelaide, SA 5005, Australia; 7 Theme of Laboratory and Community Genetics, Murdoch Childrens Research Institute, Royal Children's Hospital, Flemington Road, Parkville, VIC 3052, Australia

## Abstract

**Background:**

MicroRNAs (miRNAs) are small non-coding RNAs that can exert multilevel inhibition/repression at a post-transcriptional or protein synthesis level during disease or development. Characterisation of miRNAs in adult mammalian brains by deep sequencing has been reported previously. However, to date, no small RNA profiling of the developing brain has been undertaken using this method. We have performed deep sequencing and small RNA analysis of a developing (E15.5) mouse brain.

**Results:**

We identified the expression of 294 known miRNAs in the E15.5 developing mouse brain, which were mostly represented by *let-7 *family and other brain-specific miRNAs such as *miR-9 *and *miR-124*. We also discovered 4 putative 22-23 nt miRNAs: mm_br_e15_1181, mm_br_e15_279920, mm_br_e15_96719 and mm_br_e15_294354 each with a 70-76 nt predicted pre-miRNA. We validated the 4 putative miRNAs and further characterised one of them, mm_br_e15_1181, throughout embryogenesis. Mm_br_e15_1181 biogenesis was Dicer1-dependent and was expressed in E3.5 blastocysts and E7 whole embryos. Embryo-wide expression patterns were observed at E9.5 and E11.5 followed by a near complete loss of expression by E13.5, with expression restricted to a specialised layer of cells within the developing and early postnatal brain. Mm_br_e15_1181 was upregulated during neurodifferentiation of P19 teratocarcinoma cells. This novel miRNA has been identified as *miR-3099*.

**Conclusions:**

We have generated and analysed the first deep sequencing dataset of small RNA sequences of the developing mouse brain. The analysis revealed a novel miRNA, *miR-3099*, with potential regulatory effects on early embryogenesis, and involvement in neuronal cell differentiation/function in the brain during late embryonic and early neonatal development.

## Background

A class of small non-coding RNA (19-25 nt in length) known as microRNA (miRNA) [1-3] can exert multilevel inhibition/repression processes during post-transcriptional or protein synthesis stages [4,5]. miRNAs are transcribed in the nucleus into long polyadenylated RNAs known as primary (pri)-miRNAs that contain ~60-90 nt secondary hairpin structures termed precursor (pre)-miRNAs. The RNase III enzymes Rnasen and Dgcr8 then excise the pre-miRNA from the pri-miRNA [1,6-9]. The pre-miRNA hairpin is transported into the cytoplasm via the nuclear transport receptor, Xpo5, and further processed by another RNase III enzyme, Dicer, into a small RNA duplex containing the functional mature miRNA and a passenger strand known as miRNA star [9-11]. The majority of the miRNA star are non-functional and are rapidly degraded, but a small proportion have conserved seed regions, potentially with regulatory roles [12]. The mature miRNA forms a component of the RNA-induced silencing complexes (miRISC) and guides these complexes to mRNA targets via sequence-specific pairing between the miRNA seed sequence (the first 7 nt of the miRNA starting from position 2) and the mRNA. Typically, miRNAs guide the RISC complex to the target mRNA 3' UTR, but incidences where 5' UTR and coding-sequences were targeted have been reported [13-15]. In mammals, miRISC normally effects translational repression and, depending on the degree of miRNA:mRNA sequence complementation, can direct mRNA degradation [5,16]. Another intriguing regulatory role of miRNAs is the silencing of gene transcription which has been observed in plants [17], but has not yet been reported in the mammalian system.

Mammalian brain development requires meticulous spatio-temporal regulation of gene/protein expression, from the transcription of DNA within the nucleus to translation of mRNA in the cytoplasm [18,19]. At embryonic day 15.5 (E15.5), the mouse brain undergoes rapid cellular and anatomical changes involving neuronal migration in the cerebral cortex, proliferation of neural progenitor/stem cells at germinative zones, gliogenesis, axonogenesis and rostro-lateral to caudo-medial structure patterning [20-22]. MiRNAs play crucial roles during brain development and function. *MiR-134*, for example, is localised to the synapto-dendritic compartment of rat hippocampal neurones and has been linked to synaptic development, maturation and plasticity [23]. *MiR-9 *regulates the patterning activities and neurogenesis at the midbrain-hindbrain boundary in zebrafish [24] and *miR-124 *triggers brain-specific alternative pre-mRNA splicing leading to neuronal differentiation in the mouse [25]. MiRNAs are also associated with neurological disorders such as schizophrenia [26] and Huntington's disease [27]. To date, there are only 672 mature miRNAs in the mouse genome and 1048 in the human genome (miRBase release 16.0, September 2010) [28] in the mouse and human genomes, respectively. These figures are likely to be a gross underestimate of the actual number of miRNAs expressed. Most miRNAs are short lived, expressed in low abundance and found in specialised cell types during a specific developmental stage, and are therefore likely to remain uncharacterised due to technical limitations or the biological complexity of the tissues and cells of interest.

The emergence of next-generation sequencing technologies based on the massively parallel sequencing (MPS) concept has revolutionised the field of genomics and transcriptomics [29,30]. High-throughput generation of sequences from DNA or RNA has enabled the discovery of rare transcripts, such as alternatively spliced or fusion transcripts, as well as transcripts with low abundance [31,32]. Many next-generation sequencing datasets for small RNAs have been generated from the adult rodent and human brains [33-38]. However, to date, no small RNA profiling of the developing rodent or human brain has been performed using these methods. In this study, we performed deep sequencing of small RNAs prepared from an E15.5 mouse brain. *In silico *and laboratory based analyses led us to the discovery of 4 putative miRNAs; mm_br_e15_1181, mm_br_e15_279920, mm_br_e15_96719 and mm_br_e15_294354. Of these, mm_br_e15_1181 is novel and potentially involved in mouse embryogenesis, and brain development and function. This novel miRNA has been identified as *miR-3099*.

## Results and Discussion

### High-throughput sequencing and annotation of small RNA sequences

**Figure 1:**
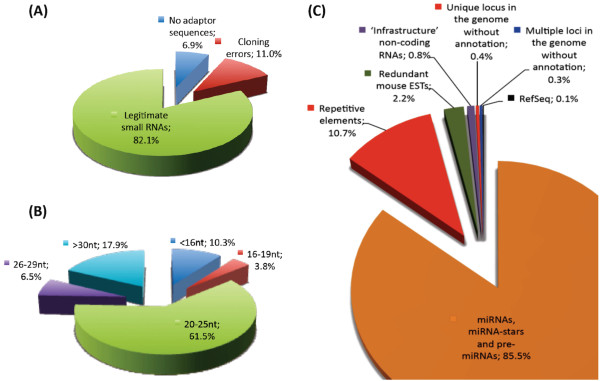
**Generation and analysis of small RNAs sequences**. There were 3,763,491 sequence reads generated. (A) Distribution of the small RNA sequences based on the analysis of 5' and 3' adaptor sequences. (B) Distribution of the small RNA sequences based on their size group. (C) Distribution of the small RNAs according to their annotations. All values presented in the figure were calculated based on the total sequence reads.

A total of 3,763,491 36 nt sequence reads were generated from a cDNA library constructed from mouse E15.5 whole brain small RNAs. The dataset was deposited into NCBI Gene Expression Omnibus GSE22653[39]. Clustering of these sequence reads revealed 413,494 unique tags (Additional file 1). Screening for adaptor sequences (both 5' and 3') using a local blastn program showed 105,993 unique tags (6.9% or 259,681 sequence reads) did not have adaptor sequences indicating contamination of larger RNA transcripts during library construction (Figure 1A). Cloning errors resulted in 40,622 unique tags (11.0% or 413,837 sequence reads) consisting of only 5'and 3' adaptor sequences. The remaining 266,879 unique tags (82.1% or 3,089,973 sequence reads) were considered legitimate as they contained partial adaptor sequences at 5' or 3' or both ends.

Of the legitimate unique tags, 59,710 (6.5% or 245,722 sequence reads) belonged to the 26-29 nt category, whereas 131,383 unique tags (61.5% or 2,314,244 sequence reads) of 20-25 nt were discovered, and therefore formed the majority of the small RNAs found in the cDNA library (Figure 1B). A total of 48,902 unique tags (3.8% or 141,783 sequence reads) were classified into the 16-19 nt category and 26,884 unique tags (10.3% or 388,224 sequence reads) of 16 nt or shorter were generated from either a pool of very small RNAs with unknown function or random RNA degradation by-products. The recent identification of tiny RNAs (~17-18 nt) shows that these small RNAs are associated with transcription initiation and splice sites specific to metazoans [40,41] suggesting that these tiny RNAs could be functional and represent another level of regulation during gene transcription in the nucleus.

Bowtie analyses, allowing only perfect matches, were performed on both the 5' and 3' end of each of the unique tags resulting in 339,201 tags (42% or 1,579,209 sequence reads) not finding a match in the mouse genome. This large proportion of unmatched unique tags included adaptors and low quality tags with errors in sequencing/base-calling. In exceptional circumstances, these unique tags could be derived from intron/exon or exon/exon boundaries, fusion transcripts or uncharacterised genomic regions. These unique tags with their corresponding sequence reads were not included for further analysis. The number of unmatched sequences varies from one study to another. Morin and colleagues reported 29-35% of their total sequence reads generated from human embryonic stem cells and embryoid bodies small RNA libraries either consisted of errors or were not perfectly matched to the human genome [42]. In a different study, deep sequencing of small RNA libraries generated from cold-treated and untreated *Brachypodium *monocot plants resulted in only 49-54% of total sequence reads matching perfectly to the genome [43]. These studies suggested that a large proportion of the total sequence reads produced by deep sequencing are discarded from further analysis due to the quality of the sequence reads and stringency imposed during sequence alignment.

A total of 74,293 unique tags (58% or 2,184,282 sequence reads) were perfectly matched to the mouse genome. Of these, 7,136 (6.2% or 234,381 sequence reads) were matched to repetitive elements, and 6,929 (0.5% or 17,853 sequence reads) were matched to 'infrastructure' non-coding RNAs such as tRNA, rRNA, scRNA, snRNA or snoRNA (Table 1; Additional files 2, 3, 4, 5, 6, 7, 8, 9, 10, 11 and 12). These unique tags and their corresponding sequence reads were also excluded from further analysis. A total of 45,623 unique tags (49.6% or 1,867,113 sequence reads) were matched to either mature miRNA, miRNA star or pre-miRNA from miRBase, 2,448 (0.1% or 2,775 sequence reads) were matched to RefSeq, 6,584 (1.3% or 48,465 sequence reads) were matched to redundant mouse EST sequences, 1,752 (0.2% or 7,656 sequence reads) mapped to a single genomic locus and 3,821 (0.2% or 6,039 sequence reads) mapped to multiple loci within the genome (Figure 1C). Intriguingly, a large number of mapped unique tags in unique genomic loci have low abundance and lack association with any known mouse mRNAs, ESTs or miRNAs suggesting that these small RNAs could be generated from specific type of cells at specific stages of development and therefore have not been characterised to date.

**Table 1:** Annotation of unique tags

	Unique tags				
			
Annotation of unique tags	22 nt of 3' end*^@^	22 nt of 5' end*^@^	Combined non-redundant^	Total combined counts^	Additional file(s)^#^
Repetitive elements	4,651	3,266	7,136	234,381	2 and 3
'Infrastructure' non-coding RNAs	6,907	30	6,929	17,853	2 and 3
miRNAs, miRNA stars and pre-miRNAs	45,623	0	45,623	1,867,113	4
RefSeq	2,431	22	2,448	2,775	5 and 6
Redundant mouse ESTs	5,954	737	6,584	48,465	7 and 8
Unique locus in the genome without annotation	1,377	439	1,752	7,656	9 and 10
Multiple loci in the genome without annotation	3,761	241	3,821	6,039	11 and 12

Total	70,704	4,735	74,293	2,184,282	

* No mismatch was allowed during Bowtie analysis.

^ Combined non-redundant values.

^@ ^Redundant values are presented. Redundant values were due to the same unique tag being analysed twice in both 5' and 3' Bowtie analysis.

^# ^Annotation of unique tags based on 3' or 5' end sequences are presented in the additional files 2, 4, 5, 7, 9, 11 and 3, 6, 8, 10, 12, respectively.

### The most abundantly expressed known miRNAs

To assess the expression of known miRNAs in the developing mouse brain at E15.5, we analysed all 294 mapped miRNAs in the dataset. Their counts ranged from 1 to 487,654 sequence reads or 0.27 to 129,575 per 1,000,000 sequence reads (CPM). The top 10% of the most abundantly expressed miRNAs are presented in Table 2 (see full list of known miRNAs in Additional file 13). The most abundantly expressed miRNA in the E15.5 developing mouse brain is *let-7c-1 *with its 7 family members (*let-7a-2*, *let-7b*, *let-7d*, *let-7e*, *let-7f-2*, *let-7g *and *let-7i*) having a combined 335,288 CPM. Our finding agrees with the first report by Lagos-Quintana and colleagues [44] regarding the high representation of *let-7 *family members in the mouse brain, which was also later found in the primate brain [45]. Despite their high level of expression in the brain, the functional role of *let-7 *in the development of the central nervous system is poorly characterised. However, the expression of *let-7 *has been associated with neural differentiation and lineage specification processes in early brain development [46].

**Table 2:** Top 10% of the most abundantly expressed known miRNAs

Small RNA ID	Accession ID	miRNA ID	Count per million	Chromosome	Start locus	Stop locus	Strand
mm_br_e15_1	MI0000559	mmu-let-7c-1	129574.91	16	77599901	77599995	+
mm_br_e15_1010	MI0000563	mmu-let-7f-2	59507.25	X	148346888	148346971	+
mm_br_e15_1001	MI0000557	mmu-let-7a-2	56984.06	9	41344798	41344894	+
mm_br_e15_10749	MI0000721	mmu-mir-9-3	27058.39	7	86650149	86650239	+
mm_br_e15_103211	MI0000137	mmu-let-7 g	25511.42	9	106081170	106081258	+
mm_br_e15_10459	MI0000561	mmu-let-7e	21824.95	17	17967315	17967408	+
mm_br_e15_1036	MI0000558	mmu-let-7b	19422.39	15	85537748	85537833	+
mm_br_e15_10	MI0000588	mmu-mir-103-2	16537.04	2	131113787	131113873	+
mm_br_e15_101787	MI0000138	mmu-let-7i	13005.48	10	122422695	122422780	-
mm_br_e15_10133	MI0000157	mmu-mir-9-2	11269.06	13	83878418	83878490	+
mm_br_e15_106	MI0000720	mmu-mir-9-1	9653.54	3	88019519	88019608	+
mm_br_e15_10266	MI0000405	mmu-let-7d	9457.18	13	48631380	48631483	-
mm_br_e15_10166	MI0000689	mmu-mir-25	7797.55	5	138606548	138606632	-
mm_br_e15_10031	MI0000155	mmu-mir-128-1	7303.33	1	130098937	130099007	+
mm_br_e15_1011	MI0000147	mmu-mir-99b	6712.12	17	17967151	17967221	+
mm_br_e15_10023	MI0000152	mmu-mir-125b-2	5810.83	16	77646517	77646588	+
mm_br_e15_1017	MI0000146	mmu-mir-99a	5567.70	16	77599180	77599245	+
mm_br_e15_13198	MI0000150	mmu-mir-124-3	3957.50	2	180628744	180628812	+
mm_br_e15_10339	MI0000144	mmu-mir-30a	3903.82	1	23279107	23279178	+
mm_br_e15_10279	MI0000165	mmu-mir-140	2629.74	8	110075143	110075213	+
mm_br_e15_1000	MI0005450	mmu-mir-181d	2452.78	8	86702614	86702686	-
mm_br_e15_10303	MI0000697	mmu-mir-181a-1	2322.84	1	139863031	139863118	+
mm_br_e15_11367	MI0000148	mmu-mir-101a	2237.28	4	101019549	101019632	-
mm_br_e15_10306	MI0000704	mmu-mir-320	2137.64	14	70843316	70843398	+
mm_br_e15_10234	MI0000684	mmu-mir-107	2068.03	19	34895176	34895263	-
mm_br_e15_10302	MI0000723	mmu-mir-181b-1	1851.47	1	139863215	139863295	+
mm_br_e15_11023	MI0000549	mmu-mir-30d	1836.86	15	68172769	68172851	-
mm_br_e15_10013	MI0000154	mmu-mir-127	1646.61	12	110831055	110831125	+
mm_br_e15_11551	MI0000729	mmu-mir-7a-2	1597.19	7	86033162	86033259	+
mm_br_e15_100	MI0000719	mmu-mir-92a-1	1563.97	14	115443648	115443728	+

Other miRNAs or miRNA families that were abundantly expressed in the E15.5 developing mouse brain include *miR-124 *(3,958 CPM), which promotes and regulates neuronal differentiation [25] and *miR-9 *(47,981 CPM), which has a role in the patterning activities and neurogenesis of the central nervous system [24]. *MiR-128 *(7,303 CPM) was highly expressed in our dataset and the finding is in agreement with a previous study [47]. Down-regulation of *miR-128 *expression has been associated with glioblastoma multiforme [48] whereas its up-regulation has been implicated with reduced neuroblastoma cell motility, invasiveness and cell growth [49]. In addition, both *miR-128 *and *miR-9 *are highly expressed in the foetal hippocampus and differentially regulated in the normal adult hippocampus as well as the hippocampus of Alzheimer's disease sufferers [50]. *MiR-125 *(5,811 CPM) and *miR-99 *(12,280 CPM) were also expressed highly in the developing mouse brain. Together with *let-7c*, both *miR-125 *and *miR-99 *are over-expressed by at least 50% in the foetal hippocampus of individuals with Down syndrome compared to age and sex matched controls suggesting that miRNAs are playing an important role in this brain region, which is pertinent for learning and long-term memory formation [51]. Interestingly, the *miR-103-2 *(16,537 CPM), *miR-107 *(2,068 CPM), *miR-181 *(6,627 CPM) and *miR-30 *(5,740 CPM) families have not previously been associated with the development of the brain, but were found to be highly expressed in our dataset. Both *miR-103 *and *miR-107 *are paralogous miRNAs and have been associated with lipid metabolism [52]. *MiR-181 *plays a crucial role in modulating haematopoietic lineage differentiation [53] whereas *miR-30 *has been strongly implicated with kidney development and nephropathies [54].

The identification of brain-related miRNAs by our deep sequencing analysis shows that the dataset is reliable not only for characterising expression profiles of known miRNAs but also for discovery of novel miRNAs. Further investigation of these miRNAs may shed light on their regulatory roles in various molecular pathways underlying the development of the embryonic brain.

### Screening and validation of putative miRNAs and pre-miRNAs

**Figure 2:**
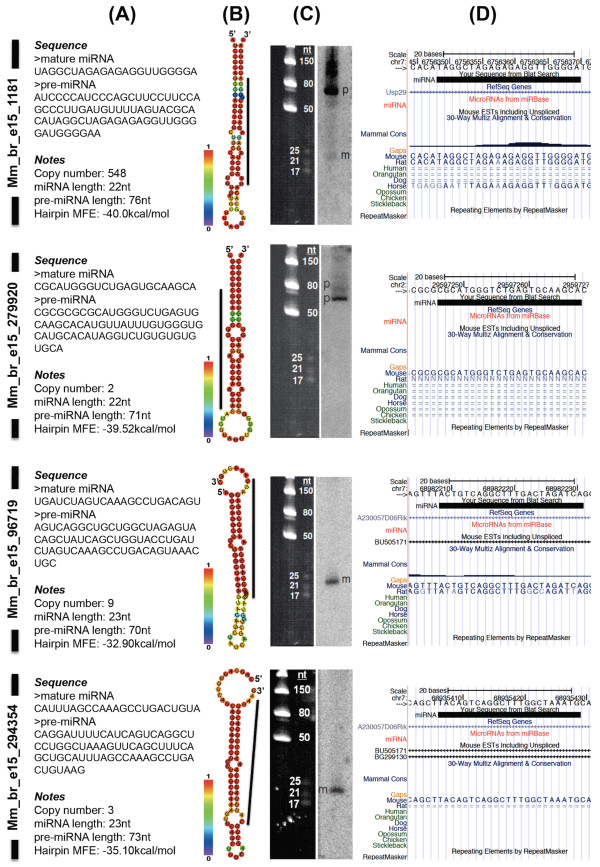
**Validated putative miRNAs**. (A) Sequences for both mature miRNA and predicted pre-miRNA. Copy number refers to the occurrences of the mature sequences in the E15.5 whole brain small RNA sequencing analysis. (B) RNAfold prediction of the stemloop hairpin structure. The colours in the vertical bar denote the base-pairing probability between two nucleotides within the structure. The black line located next to the hairpin structure denotes the position of the small RNA within the pre-miRNA. (C) Small RNA northern analysis using radiolabeled oligonucleotide probes. 'p' and 'm' refer to pre-miRNA and mature miRNA, respectively. Four independent small RNA northern blots were used to validate the putative miRNA. After hybridization and washing steps, mm_br_e15_1181 blot was exposed to phosphor screen for 1 day whereas the other 3 blots for mm_br_e15_279920, mm_br_e15_96719 and mm_br_e15_294354 were exposed for 8 days. (D) Mapping of the mature miRNA to the mouse genome and other corresponding features such as RefSeq genes, miRNAs from miRBase, mouse ESTs, mammalian conservation information and repeating elements.

To identify putative miRNAs, we analysed unique tags with a single match to the genome that were annotated as matched to RefSeq or redundant mouse EST sequences or were without annotation. A total of 10,784 unique tags (1.6% or 58,896 sequence reads) were selected under these criteria. We included all sequences with 1-2 counts into the analysis because we had found 34 known miRNAs residing in a similar range of expression within the dataset (see Additional File 13), suggesting some of the single count unique tags might be true positives. Pre-miRNA sequences were predicted using the RNA22 program, a pattern-based method reported previously [55]. The program predicted 8 putative miRNAs with pre-miRNA sequences; mm_br_e15_1181, mm_br_e15_279920, mm_br_e15_96719, mm_br_e15_294354, mm_br_e15_276138, mm_br_e15_331608, mm_br_e15_255873 and mm_br_e15_363469 (see Additional File 14). The resulting candidate pre-miRNA sequences were subjected to hairpin structure or fold prediction using the RNAfold program [56]. Of all the candidate putative miRNAs, only 4 fulfilled the criteria outlined for mature miRNA and pre-miRNA [2]. These were mm_br_e15_1181 (chr7:6756349-6756370), mm_br_e15_279920 (chr2:29597247-2959768), mm_br_e15_96719 (chr7:68982209-68982231), and mm_br_e15_294354 (chr7:68935407-68935429) which featured a 22-23nt mature miRNAs and a 70-76nt predicted pre-miRNAs (Figure 2A B and 2D). The other 4 putative miRNAs, mm_br_e15_276138, mm_br_e15_331608, mm_br_e15_255873 and mm_br_e15_363469 contained a large internal loop, branching stem or oversized pre-miRNA structural properties (see Additional file 14). These putative miRNAs were excluded from further analysis.

Mm_br_e15_1181 was matched to the second intron of the ubiquitin specific peptidase 29 (*Usp29*) gene. Mm_br_e15_279920 was matched to a single locus within the mouse genome without any annotations, whereas both mm_br_e15_96719 and mm_br_e15_294354 miRNAs were matched to two different introns of the same EST, BU505171. We performed a small RNA northern analysis on the E15.5 whole brain small RNAs to validate all the 4 predictions. We also included mm_br_e15_276138, mm_br_e15_331608, mm_br_e15_255873 and mm_br_e15_363469 in our northern analysis to serve as negative controls. The analysis confirmed all 4 predictions at the mature miRNA level for mm_br_e15_1181, mm_br_e15_96719 and mm_br_e15_294354, and at the pre-miRNA level for mm_br_e15_1181 and mm_br_e15_279920 (Figure 2C). As expected, the northern analysis of negative controls showed no detectable signals for mm_br_e15_276138 and mm_br_e15_363469, and multiple bandings for mm_br_e15_331608 and mm_br_e15_255873, signifying random by-products due to RNA degradation (see Additional file 14). Depending on the biological context of the assessed tissue, miRNA may be preserved or accumulated at the pre-miRNA level due to specific factors such as the activity levels of dicer, argonaute or nuclear export receptors [57-59]. Therefore, we considered the existence of these small RNAs validated when either the mature or precursor miRNA with specific size was detected using the northern analysis.

Further analysis using the University of California, Santa Cruz (UCSC) genome browser [60] showed that mm_br_e15_1181 was mapped to a region within the mouse genome that is homologous to the rat and horse genomes. Other putative miRNAs were mapped either to a region specific to the mouse genome (mm_br_e15_294354) or a region homologous to the rat only (mm_br_e15_279920 and mm_br_e15_96719) (Figure 2D). By using both the full-length and seed sequences of all the 4 putative miRNAs, we performed homology searches against all the known miRNA sequences and were unable to find any orthologous miRNAs, indicating that these putative miRNAs could be specific to the mouse or rat especially mm_br_e15_1181 and mm_br_e15_96719. Sequence conservation of miRNAs is relatively common among vertebrates as well as invertebrates. For example *miR-263 *(consisting of *miR-263a *and *miR-263b*) and *miR-183 *(consisting of *miR-96, miR-182 *and *miR-183*) families are found in many organisms including human, mouse, chicken, zebrafish, frog, worm and fruit fly, with high sequence and expression profile similarity particularly in sensory organs [61,62]. However, lack of sequence homology among miRNAs from different organisms does not negate the possibility of functional conservation among them. For example, both *lin-4 *and *let-7 *target multiple sequence motifs at the 3' UTR of *Caenorhabditis elegans *hunchback homolog mRNA, *hbl-1*, and regulate its expression in the ventral nerve cord neurones [63]. In addition, different miRNAs with similarity at the seed region may exert the same effect on a same mRNA. *Drosophila *bearded (*Brd*) gene has motifs that are complementary to two different miRNAs, *miR-4 *and *miR-79*, which bear the same seed sequence. Both the miRNAs target the motifs based entirely on the seed sequence with little or no base-pairing to the 3' region [64]. Although this phenomenon is rare across different organisms, it proves that functional conservation between non-conserved miRNAs may lie within the seed region alone.

### Mm_br_e15_1181 biogenesis is Dicer1-dependent

**Figure 3:**
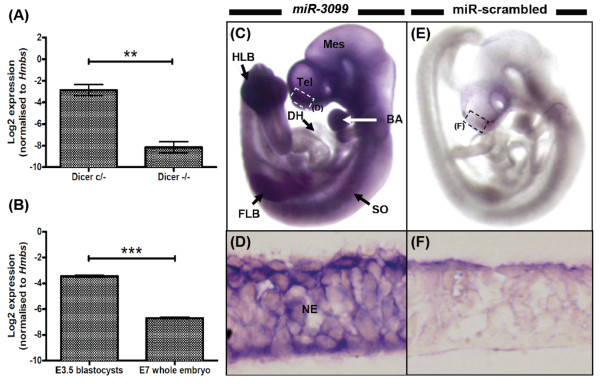
**Expression profiling of *miR-3099 *novel miRNA in mouse embryonic stem cells (with conditional allele for Dicer), E3.5 blastocysts, E7 and E9.5 embryos**. (A) Stemloop RT-qPCR analysis of *miR-3099 *novel miRNA in mouse embryonic stem (mES) cells with conditional allele for *Dicer1*. mES cells with and without Dicer1 activity are denoted by Dicer c/- and Dicer -/-, respectively (n = 3 per group). (B) Expression of *miR-3099 *in E3.5 blastocysts (n = 14; pooled) and the E7 whole embryo (n = 3). (C-F) Whole mount ISH of E9.5 embryos using DIG-labeled LNA probes for *miR-3099 *(C and D) (n = 3) and miR-scrambled (E and F) (n = 2). Cryosection of the stained embryos shows expression of *miR-3099 *in the neuroepithelium of the telencephalon (D, inset in C). BA = branchial arches, DH = developing heart, FLB = forelimb bud, HLB = hindlimb bud, Mes = mesencephalon, NE = neuroepithelium, SO = somite, Tel = telencephalon. The mean ± SE for each tissue is presented in the bar graphs. Asterisks denote the statistical significance level at *P *< 0.01 (**) and *P *< 0.001 (***) based on the one-way ANOVA test (see Additional file 14 for analysis details).

Of the 4 putative miRNAs, we selected mm_br_e15_1181 for further characterisation due to its high copy number. First, we evaluated mm_br_e15_1181 expression in mouse embryonic stem (mES) cells, with and without Dicer1 enzyme activity using the stemloop RT-qPCR technique (Figure 3A). Mm_br_e15_1181 was expressed in mES cells with Dicer1 activity, however its expression was not detected or was weak in cells lacking Dicer1 activity confirming that mm_br_e15_1181 biogenesis is Dicer1-dependent (*P *< 0.01). The evaluation of Dicer1-dependency using the mES cell model is limited to miRNAs that are expressed in this cell type. It is worth noting that Dicer1-dependency is not a definitive property for defining mm_br_e15_1181 as a novel miRNA because endogenous small siRNAs are also subjected to the same dicing mechanism in the cytoplasm [1]. A recent study reported the Dicer1-independent biogenesis of *miR-451*, in which the catalytic activity of Argonaute2 was responsible for the *pre-mir-451 *hairpin cleavage process [65].

In this study, we used a number of validation analyses for mm_br_e15_1181: Dicer1-dependence, pre-miRNA structure prediction and northern analysis to define mm_br_e15_1181 as a novel miRNA. This novel miRNA has been identified as *miR-3099*.

### Expression profiling of *miR-3099 *throughout embryogenesis

The expression of *miR-3099 *in mES cells led us to hypothesize that this miRNA may play a role in early embryogenesis and therefore we characterised its expression profile throughout development. Using stemloop RT-qPCR, we showed that *miR-3099 *was expressed in E3.5 blastocysts (Figure 3B). The expression of *miR-3099 *reduced (by ~9-fold; *P *< 0.001) as the blastocysts developed into an early stage embryo at day 7 (E7), suggesting that *miR-3099 *was either expressed in a spatially restricted manner or generally down-regulated at this stage. To specifically locate the expression of *miR-3099 *during embryogenesis, we performed whole mount *in situ *hybridisation on E9.5 embryos (n = 3) and showed that *miR-3099 *was expressed throughout the embryo with the exception of the developing heart (Figure 3C). Stronger expression was observed in the telencephalon, somites, branchial arches, and both forelimb and hindlimb buds. Cross sectional analysis of the telencephalon confirmed that *miR-3099 *was expressed in the neuroepithelium (Figure 3D). Whole mount ISH analysis on embryos of the same age was performed using *miR-scrambled *LNA probe to serve as the background control (n = 2) (Figure 3E &3F).

**Figure 4:**
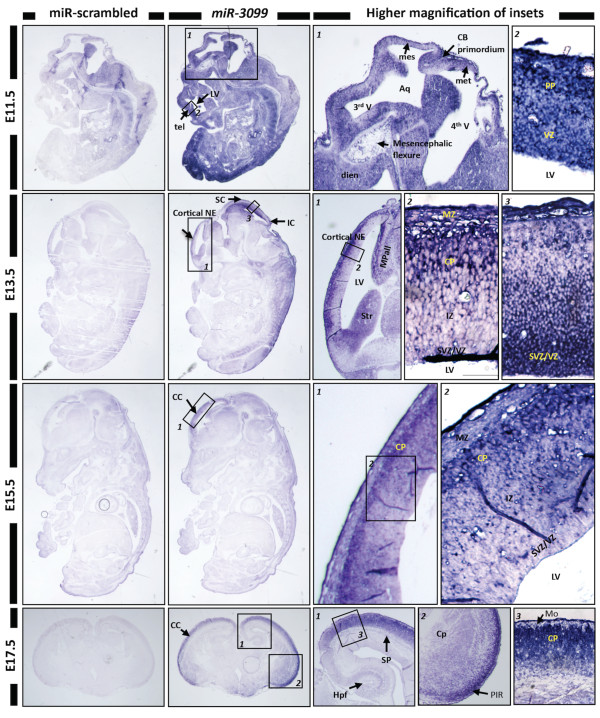
**Expression profiling of *miR-3099 *novel miRNA in E11.5-E15.5 whole embryos and the E17.5 whole brain**. *In situ *hybridisation analysis using LNA probes for miR-scrambled and *miR-3099 *was performed on E11.5-E15.5 developing embryos and E17.5 whole brain paraffin sections. Strong expression of *miR-3099 *was detected in the E11.5 embryo. From E13.5 onwards, the expression was retained only in the neuroepithelium (NE) or cerebral cortex (CC). Under high magnification, *miR-3099 *was found to express specifically in the preplate (PP) of telencephalon (tel) (E11.5), cortical plate (CP) of the CC (E13.5-E17.5) and the germinal layer of mesencephalon (mes) (E11.5-E13.5). Aq = aqueduct, CB = cerebellum, Cp = caudo-putamen, dien = diencephalon, Hpf = hippocampal formation, IC = inferior colliculus, IZ = intermediate zone, LV = lateral ventricle, met = metencephalon, Mo = molecular layer, MPall = medial pallium (hippocampal allocortex), MZ = marginal zone, PIR = piriform cortex, SC = superior colliculus, SP = subplate, Str = striatum, SVZ = subventricular zone, V = ventricle.

To evaluate the expression profile of *miR-3099 *in the later stages of embryogenesis, we performed section ISH. Section ISH of the E11.5 whole embryos showed that *miR-3099 *was expressed throughout the embryo, especially in the preplate of the telencephalon, somites and hindlimb region (Figure 4). By E13.5, *miR-3099 *expression was restricted to the cortical plate of the cortical neuroepithelium, striatum, medial pallium (hippocampal allocortex) and subventricular/ventricular zone of the superior and inferior colliculi. In E15.5 embryos, *miR-3099 *expression was observed primarily in the cortical plate of the cerebral cortex. In E17.5 whole brains, *miR-3099 *expression was prominent in the cortical plate, piriform cortex and at lower levels, in the hippocampal formation. Embryo-wide expression of *miR-3099 *during early embryogenesis suggests a pan-regulatory role, possibly functioning as a 'housekeeping' miRNA in basic cellular processes. This feature has been described in a few clusters of miRNAs expressed in the mouse retina, brain and heart [66]. Many miRNAs have ubiquitous expression patterns and their function remains unclear as they may have roles in subtle miRNA networks, which exert combinatorial effects during development [67,68]. Contrasting with the almost ubiquitous expression profile in early development, *miR-3099 *was not detected in a few regions such as the E9.5 developing heart and the ventricular zone of the telencephalon/developing cerebrum. This suggests that the function of *miR-3099 *may be tissue or cell-specific, especially after E11.5, this warrants further characterisation.

**Figure 5:**
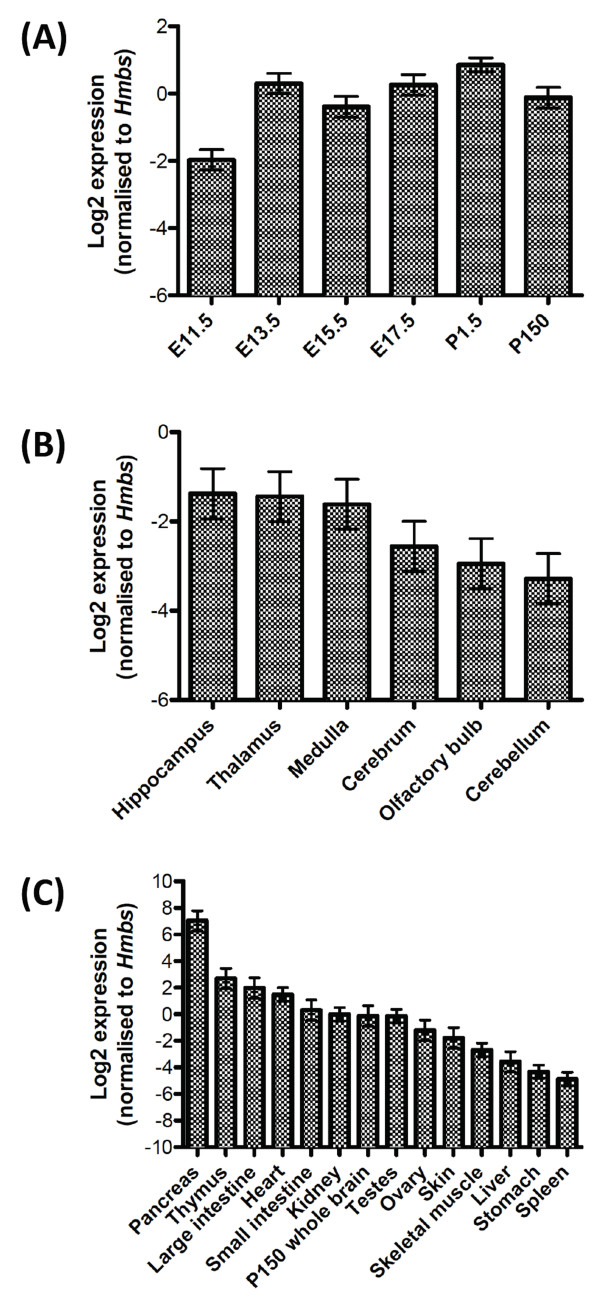
**Expression profiling of *miR-3099 *novel miRNA in the whole brain of different developmental stages, different adult mouse brain regions and organs**. Stemloop RT-qPCR analysis of *miR-3099 *in E11.5-P150 whole brain (A), brain regions in P150 whole brain (B) (n = 2 for each group) and various mouse organs harvested from P150 adult mouse (C) (n = 2 for all except P150 whole brain, skeletal muscle, spleen, stomach and testes, where n = 3). The mean ± SE for each organ is presented in the bar graphs. The one-way ANOVA test is significant at *P *< 0.05 for (A), not significant for (B) and *P *< 0.001 for (C) (see Additional file 14 for analysis details).

We also performed stemloop RT-qPCR expression analysis of *miR-3099 *in various regions of the mouse brain and organs. Using the mouse whole brain, there was a significant difference (*P *= 0.02) in the *miR-3099 *expression among E11.5, E13.5, E15.5, E17.5, postnatal day (P) 1.5 and P150 samples (Figure 5A). *MiR-3099 *expression was found to be increased after E11.5 and was maintained in postnatal day 1.5 (P1.5) and P150 whole brains. The qPCR analysis supports the previous section ISH analysis. No significant differences (*P *= 0.45) in *miR-3099 *expression were observed among cerebellum, cerebrum, hippocampus, medulla, olfactory bulb and thalamus (Figure 5B). When we compared the expression of *miR-3099 *in various adult mouse organs to the P150 whole brain, we found significant differences in the expression levels among the organs (*P *< 0.001) (Figure 5C). *MiR-3099 *was found to be expressed at the highest level in the pancreas, followed by the thymus, large intestine, heart, small intestine, kidney, brain, testis, ovary, skin, skeletal muscle, liver, stomach and spleen. Similar to the embryonic expression profiles, the diverse expression profile of *miR-3099 *in multiple organs of the adult mouse further supports a widespread role in the development and function of these organs.

### Expression of *miR-3099 *is upregulated in differentiating neuronal/glial cells

**Figure 6:**
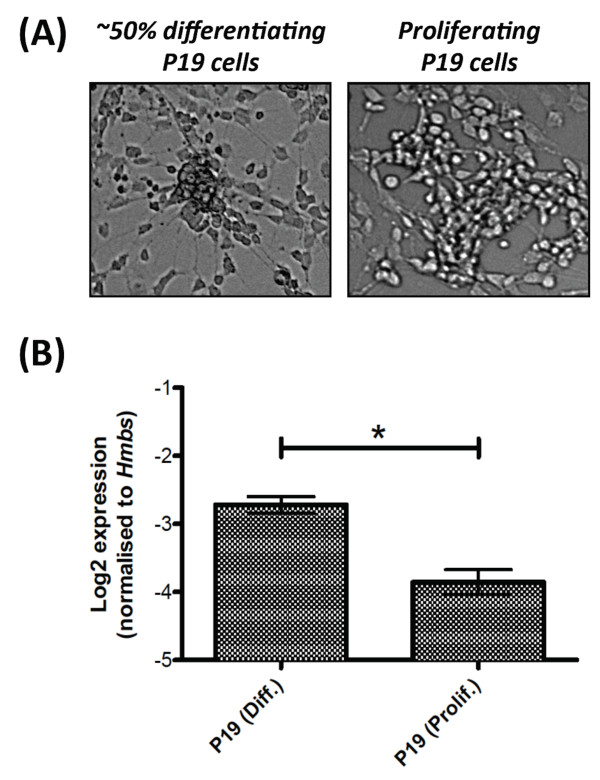
**Expression of *miR-3099 *in P19 teratocarcinoma cells**. (A) Phase contrast micrographs of differentiating and proliferating P19 cells. (B) Stemloop RT-qPCR analysis of *miR-3099 *expression in differentiating (diff.) (n = 3) and proliferating (prolif.) (n = 2) P19 cells. The mean ± SE for each cell type is presented in the bar graph. The asterisk (*) denotes statistical significance at *P *< 0.05 based on the one-way ANOVA test (see Additional file 14 for analysis details).

Expression of *miR-3099 *was observed in the preplate of the E11.5 telencephalon and later in the cortical plate of the E13.5-E17.5 cerebral cortex, by which time the majority of the cells in these structures are committed to their respective neuronal lineages. This finding further suggests that *miR-3099 *may play an important regulatory role during neurogenesis or in neuronal function. To further test this idea, we used P19 teratocarcinoma cells as an *in vitro *model. Upon retinoic acid induction and under reduced serum concentration, P19 cells differentiate into glutamatergic and glutamate-responsive neurones, glial and fibroblast-like cells [69-72]. We analysed the expression level of *miR-3099 *in P19 cells (Figure 6A) and found a statistically significant (*P *= 0.04) ~2-fold upregulation of *miR-3099 *in ~50% differentiated P19 cells compared to the proliferating cells (Figure 6B). Various miRNAs have been found to be upregulated during neural differentiation and some of their expression could be negatively regulated by important transcription factors such as Oct4 and Sox2, the expression levels of which gradually diminish as cells differentiate into neurones [73]. Therefore, increased *miR-3099 *expression during P19 differentiation raises the possibility that this miRNA may have a functional role during neural differentiation or neuronal cell function.

## Conclusions

In this study, we have reported the first deep sequencing analysis of small RNAs of a developing mouse brain. We have identified and validated 4 putative miRNAs from the analysis and further characterised one of them, *miR-3099*, during embryogenesis. A significant finding of the study was the embryo-wide expression profile of *miR-3099 *in mid-gestation embryos, which became restricted to the central nervous system, suggesting a role for this miRNA in neural differentiation or function.

## Methods

### Animals and dissections

The Melbourne Health Animal Ethics Committee and the University of Adelaide Animal Ethics Committee approved procedures involved in the breeding and handling of animals. Mice were housed under a 12-hour light and 12-hour dark cycle with access to unlimited food and water. Mice were culled by CO_2 _inhalation and all dissections of mouse embryos, brains and organs were carried out according to the methods described previously [18].

### Deep sequencing and analysis

Total RNA was isolated from a whole brain dissected from an E15.5 embryo of C57BL/6 background using TRIzol reagent (Invitrogen) according to the manufacturer's protocol. Small RNAs with sizes ranging from 16-30nt were isolated from 10 μg total RNA using polyacrylamide gel electrophoresis. The complementary small RNA library was constructed using the Small RNA Sample Prep Kit version 1.0 (Illumina) according to the manufacturer's protocol with 5'-GTTCAGAGTT CTACAGTCCG ACGATC-3' and 5'-TCGTATGCCG TCTTCTGCTT GT-3' adapters at the 5' and 3' ends, respectively. Sequencing was carried out using a Genome Analyzer II (Illumina). Image data was generated by the Genome Analyzer II and was processed using the Illumina pipeline software (Pipeline version 1.0 was used for the FASTQ data). This consists of an image analysis module (Firecrest), followed by basecalling using the BUSTARD module and finally production of a data file in FASTQ format using the GERALD module.

### Sequence annotation pipeline

The FASTQ data was ranked according to decreasing abundance of the unique tags. This file was created using a PERL script in Linux without taking into consideration any filters (adapter sequences) or quality. A file with unique tags and their corresponding counts was generated. All unique tags (including those with a single count) were mapped to the NCBI Mouse Assembly Build 37.1 using the Bowtie program [74]. Two sets of alignments were carried out: one stripping off 14 bases from the 5' end of unique tags and the other stripping off 14 bases from the 3' end. In both alignments, no mismatches are allowed and unique tags that hit more than one locus within the mouse genome were discarded. Unique tags with a single hit within the genome were further annotated using various databases such as RepeatMasker (analysis was performed on NCBI Mouse Assembly build 37.1 and the output was downloaded from UCSC genome browser on the 28^th ^of November, 2008), mouse RefSeq in release 32, mouse miRNA in miRBase release 12.0 and redundant mouse EST database (downloaded from UCSC mm9 on 27^th ^January, 2009).

### Identification of candidate novel miRNAs

Unique tags that mapped to a genomic locus with a RefSeq, redundant EST or no annotations were subjected to pre-miRNA prediction using the RNA22 program [55]. Sequences encompassing 100- to 200-nt upstream and downstream of these unique sequences were used to predict any potential pre-miRNAs with hairpin structures. The minimum number of patterns that should support a pre-miRNA before it can get reported was set to 60, and the minimum and maximum pre-miRNA lengths were set to 60nt and 150nt, respectively. All predicted pre-miRNA sequences based on these settings were used to determine the hairpin fold structure using RNAfold program [56]. The predicted hairpin fold structure with the lowest minimum free energy (MFE) (cut off at -30 kcal/mol or lower) and conforming to the annotation criteria for pre-miRNA [2] was selected as the final predicted pre-miRNA. Briefly, the predicted precursor structure must be between 60-80 nt in size and must not have a large internal loop or any asymmetric bulges. The predicted pre-miRNA must contain the aligned unique sequence within one arm of the hairpin and include at least 16 bp from the 5' end of the unique sequence and the other arm of the hairpin.

### Small RNA northern analysis

Eight blots were prepared from four independent E15.5 whole brains. Approximately 30 μg of total RNA was denatured in 1X Ambion Gel Loading Buffer II (Ambion^®^) at 85°C for 3 minutes. RNAs were electrophoresed in 15% acrylamide/urea gels (48% (w/v) urea, 15% (v/v) acrylamide, 0.05% (w/v) ammonium persulfate and 0.1% (v/v) tetramethylethylenediamine prepared in 1X TBE) in 1X TBE buffer at 300 V for 90 minutes. Separated small RNAs in the gel were then transferred onto Hybond-N+ nylon membrane (GE Healthcare) using Trans-Blot^® ^SD Semi-Dry Electrophoretic Transfer Cell (Bio-Rad) at a constant 0.4 V for 45 minutes. The pre-hybridisation step was carried out in Amersham Rapid-hyb™ Buffer (GE Healthcare) with 100 μg/ml of herring sperm DNA (Promega) at 42°C for 1 hour and was followed by the hybridisation step. The same pre-hybridisation solution was used for hybridisation with addition of 2 × 10^6 ^dpm/ml labelled probe prepared using 20 U of T4 Polynucleotide Kinase (Promega) in 1X kinase buffer (Promega) and 50 pmol of [γ-^32^P]-dATP (GE Healthcare) (3000 Ci/mmol). Hybridisation was carried out for 18 hours and filters were washed in 5 × SSC with 0.1% (w/v) sodium dodecyl sulfate (SDS) (20 minutes at 37°C) followed by 1 × SSC with 0.1% (w/v) SDS and 0.2 × SSC with 0.1% (w/v) SDS (15 minutes each time at 65°C until a clean background signal was obtained). The membrane was exposed to a storage phosphor screen in a cassette at room temperature for 1 day for *miR-3099 *blot and 8 days for other blots before scanned using Typhoon™ 9400 (GE Healthcare).

### Stemloop RT-qPCR

Reverse transcription of the small RNA was performed based on modified methods [75,76]. cDNA was synthesised from 150 ng-2.5 μg of small RNA enriched total RNA using 0.05 μM of an in-house designed stem loop primer (5'-GTTGGCTCT GGTAGGATG CCGCTCTCA GGGCATCCT ACCAGAGCCA AACTCCCCA-3', GeneWorks), and the Superscript^® ^III Reverse Transcriptase Kit (Invitrogen) with modifications to the manufacturer's protocol. The stem loop primer was added after a denaturation step at 65°C for 5 minutes. The last 6nt at the 3' end of the stem loop primer complements the last 6nt of the 3' end of *miR-3099 *small RNA. The stem loop RT primer contains a target site for a universal reverse primer (5'-GTAGGATGCC GCTCTCAGG-3', GeneWorks) and a target site for UniversalProbe Library (UPL) Probe #21 (Roche Diagnostics), which were used in subsequent cDNA amplification processes together with a specific forward primer for *miR-3099 *(5'-CGCGTAGGCT AGAGAGAGGT-3', GeneWorks). Briefly, cDNA synthesis was performed at 16°C for 30 minutes followed by 60 cycles of 20°C for 30 seconds, 42°C for 30 seconds and 50°C for 1 second. A final incubation at 75°C for 15 minutes was performed to inactivate the reverse transcriptase enzyme.

Prior to qPCR, pre-PCR of *miR-3099 *was performed in a 10 μl reaction volume containing 1X LC480 Probe Master mix (Roche Diagnostics), 50 nM of each forward and universal reverse primers and 0.2X of synthesised cDNA. Pre-PCR was initially carried out at 95°C for 10 minutes, 55°C for 2 minutes and 75°C for 2 minutes and followed by 14 additional cycles of 95°C for 15 seconds and 60°C for 4 minutes. After pre-PCR, 0.01X of amplicons were used for qPCR.

QPCR was carried out in 10 μl reaction volume using 1X LightCycler 480 (LC480) Probe Master mix (Roche Diagnostics), 0.1 μM of a relevant Universal ProbeLibrary probe (Roche Diagnostics), 0.25 μM of each forward and reverse primers and 1 μl of 0.1X of synthesised cDNA. Reactions were prepared in 384-well plates and RT-qPCR was performed using a LightCycler^® ^480 Real Time PCR System instrument (Roche Diagnostics). QPCR was performed with an initial denaturation at 95°C for 10 minutes followed by 45 cycles at 95°C for 10 seconds, 60°C for 30 seconds and 72°C for 10 seconds, and a final step at 40°C for 1 second.

Real-Time amplification signals were acquired during the elongation step and recorded live using LightCycler^® ^480 Software version 1.5 (Roche Diagnostics). The cycle threshold or crossing point (Cp) from each signal was calculated based on the Second Derivative Maximum method [77]. A 4-data point standard curve was constructed using serially diluted pooled cDNAs for each primer set used in qPCR in each run. The standard curve was used to determine the PCR efficiency and reproducibility of each PCR system. The *Hmbs *gene was used as reference gene normalisation according to the method as described [18].

### Statistical analysis

Two or three independent biological replicates were used for each tissue/organ in each experiment. Two qPCR experiments were performed on the tissue of each biological replicate. The qPCR results were normalized to *Hmbs*, and those that were not outliers, log_2 _transformed and then averaged to give the expression data for the biological replicate. One-way ANOVA was used to compare the expression levels among the tissues. A *P *value of <0.05 was considered statistically significant. Where significant differences were detected among the tissues the least significant difference(s) (LSD) were provided with the analysis (see Additional file 14 for analysis details).

### Locked Nucleic Acids - *In situ *hybridisation

Paraffin embedded sections (8 μm) were used for LNA-ISH. Sections were de-paraffinised with washes in xylene (3× for 5 minutes each) and hydrated in a series of ethanol concentrations into RNase-free water. Subsequently, sections were fixed in 4% (w/v) PFA (pH7.0) in 1X PBS (10 minutes) followed by Proteinase K digestion (6.7 μg/ml of Proteinase K, 50 mM of Tris HCl pH7.5, 5 mM of EDTA) for 30 minutes, re-fixed in 4% (w/v) PFA in 1X PBS for 5 minutes and acetylated (0.1 M of triethanolamine, 0.178% (v/v) of concentrated HCl and 0.25% (v/v) of acetic anhydride) for 10 minutes. Between each step, sections were washed multiple times using 1X PBS.

The pre-hybridisation step was carried out in a humidified chamber (50% (v/v) formamide, 5X sodium chloride/sodium citrate, SSC) at 60°C. Amersham Rapid-hyb™ Buffer (GE Healthcare) was used for pre-hybridisation with additional *Escherichia coli *tRNA (Sigma Aldrich) and Herring Sperm DNA (Promega) to a final concentration of 100 μg/ml each. After 1-2 hours of pre-hybridisation, custom-made *Sox4_sir3 *LNA probes (Cat. no: EQ-70537, Exiqon) were added to the buffer to give a concentration of 0.020 pmol/μl. Hybridisation was carried out in the oven for 16-20 hours.

After the hybridisation step, sections were washed in 5 × SSC (20 minutes at hybridisation temperature) followed by 0.2 × SSC (3 hours at hybridisation temperature). Sections were then rinsed in fresh 0.2 × SSC for 5 minutes and in pre-blocking buffer (0.1 M of Tris HCl pH7.5, 0.15 M of NaCl and 240 μg/ml of levamisole) for a further 5 minutes. In a humidified chamber, sections were blocked in 20% (v/v) foetal calf serum (Sigma Aldrich) and 2% (w/v) blocking powder (Roche Diagnostics) in maleate buffer for 1 hour. After blocking, sections were incubated with 0.0002X (0.00015 U) anti-DIG antibody with alkaline phosphatase, Fab fragments (Roche Diagnostics) in blocking buffer for 16 hours in the dark. Subsequently, sections were washed in NTMT buffer (3× for 10 minutes each: 0.1 M Tris HCl pH9.5, 0.1 M NaCl, 0.05 M MgCl_2_, 1% (v/v) Tween-20 and 240 μg/ml levamisole) and then with nitro blue tetrazolium chloride (NBT)/5-Bromo-4-chloro-3-indolyl phosphate, toluidine salt (BCIP) colour reaction (0.375 mg/ml of NBT and 0.188 mg/ml of BCIP in NTMT buffer) for 3 hours to 5 days. After the colour reaction step, sections were washed with Tris EDTA buffer pH8.0 (0.01 M of Tris HCl pH7.5 and 0.001 M EDTA pH8.0) for 10 minutes and were mounted in Entellan^® ^media (ProSciTech).

### P19 teratocarcinoma cells

Propagation and differentiation of P19 cells were carried out according to protocols previously described [18,78].

### Mouse embryonic stem (mES) cells with Dicer1^c ^conditional allele

Mouse embryonic stem (mES) cells with Dicer1 activity were of a line heterozygous for a conditionally mutant *Dicer1 *allele (*Dicer1*^*c*^) and a null *Dicer1 *allele (*Dicer1*^-^), these genetic modifications have been previously described [79]. mES cells without Dicer1 activity were produced by transient transfection of this *Dicer1*^*c*^^/- ^line with Cre recombinase to produce *Dicer1*^-/- ^subclones (JRM and DMM, unpublished data). The mES cells were propagated as previously described [80].

### Mouse E3.5 blastocysts

C57BL/6 females of 3-4 weeks of age were superovulated using 5IU of Folligon (PMSG) followed by 5IU of Chorulon (HCG) 47.5 hours later and mated with B6D2F1 entire stud males. Microdrop culture dishes were set up to equilibrate in 37°C, 5% CO_2 _incubator 4 hours prior to culture. KSOM (Millipore) media was used in 20 μl droplets in a 35 mm dish, overlaid with Embryo Tested Mineral Oil (Sigma). Superovulated female mice were sacrificed after 2.5 days of superovulation induction and mating, and oviducts were collected into M2 handling media (Millipore). Oviducts were flushed using M2 media, a blunt 30G needle and a1ml syringe. Morulae were collected and cultured in pre-equilibrated KSOM. Blastocysts were collected from culture a day later under a dissecting microscope. These were considered E3.5 blastocysts.

## Authors' contributions

KHL, JRR, JMR and DLA participated in the small RNA sequence analysis and annotations. KHL, PJB and CNH supervised, designed and carried out the qPCR analysis. KHL and TD performed the small RNA northern analysis. PQT supervised whereas KHL and PSC performed the LNA-ISH analysis. SP performed the procedures for superovulation and mating of mice, cultured the morulae and provided the blastocysts for the expression analysis. DMM and JRM cultured and provided the mES cells with Dicer1 conditional allele. KHL drafted the manuscript. CNH, PQT, DLA and HSS conceived of the study, and participated in its design and coordination. All authors read and approved the final manuscript.

## Supplementary Material

Additional file 1 **List of 413,494 unique tags**. Click here for file

Additional file 2 **List of unique tags mapped to repetitive elements and ncRNAs based on the 3' end sequences**. Click here for file

Additional file 3 **List of unique tags mapped to repetitive elements and ncRNAs based on the 5' end sequences**. Click here for file

Additional file 4 **List of unique tags mapped to miRNAs, pre-miRNAs or miRNA-star based on 3' end sequences**. Click here for file

Additional file 5 **List of unique tags mapped to RefSeq sequences based on 3' end sequences**. Click here for file

Additional file 6 **List of unique tags mapped to RefSeq sequences based on 5' end sequences**. Click here for file

Additional file 7 **List of unique tags mapped to redundant mouse ESTs based on 3' end sequences**. Click here for file

Additional file 8 **List of unique tags mapped to redundant mouse ESTs based on 5' end sequences**. Click here for file

Additional file 9 **List of unique tags mapped to a single locus in the mouse genome based on 3' end sequences**. Click here for file

Additional file 10 **List of unique tags mapped to a single locus in the mouse genome based on 5' end sequences**. Click here for file

Additional file 11 **List of unique tags mapped to multiple loci in the mouse genome based on 3' end sequences**. Click here for file

Additional file 12 **List of unique tags mapped to multiple loci in the mouse genome based on 5' end sequences**. Click here for file

Additional file 13 **List of known miRNAs in the E15.5 developing mouse brain**. Click here for file

Additional file 14 **Supplementary figures and data for statistical analysis**. Click here for file
