# Supplementary material for: Deep sequencing analysis of the developing mouse brain reveals a novel microRNA
Source: BMC Genomics. 2011 Apr 5;12:176. doi: 10.1186/1471-2164-12-176 (PMC3088569; doi:10.1186/1471-2164-12-176)
Supplement: Additional file 14 — Supplementary figures and data for statistical analysis. [file 1471-2164-12-176-S14.DOC]

**Additional file 14**

**Supplementary figures and data for statistical analysis.**


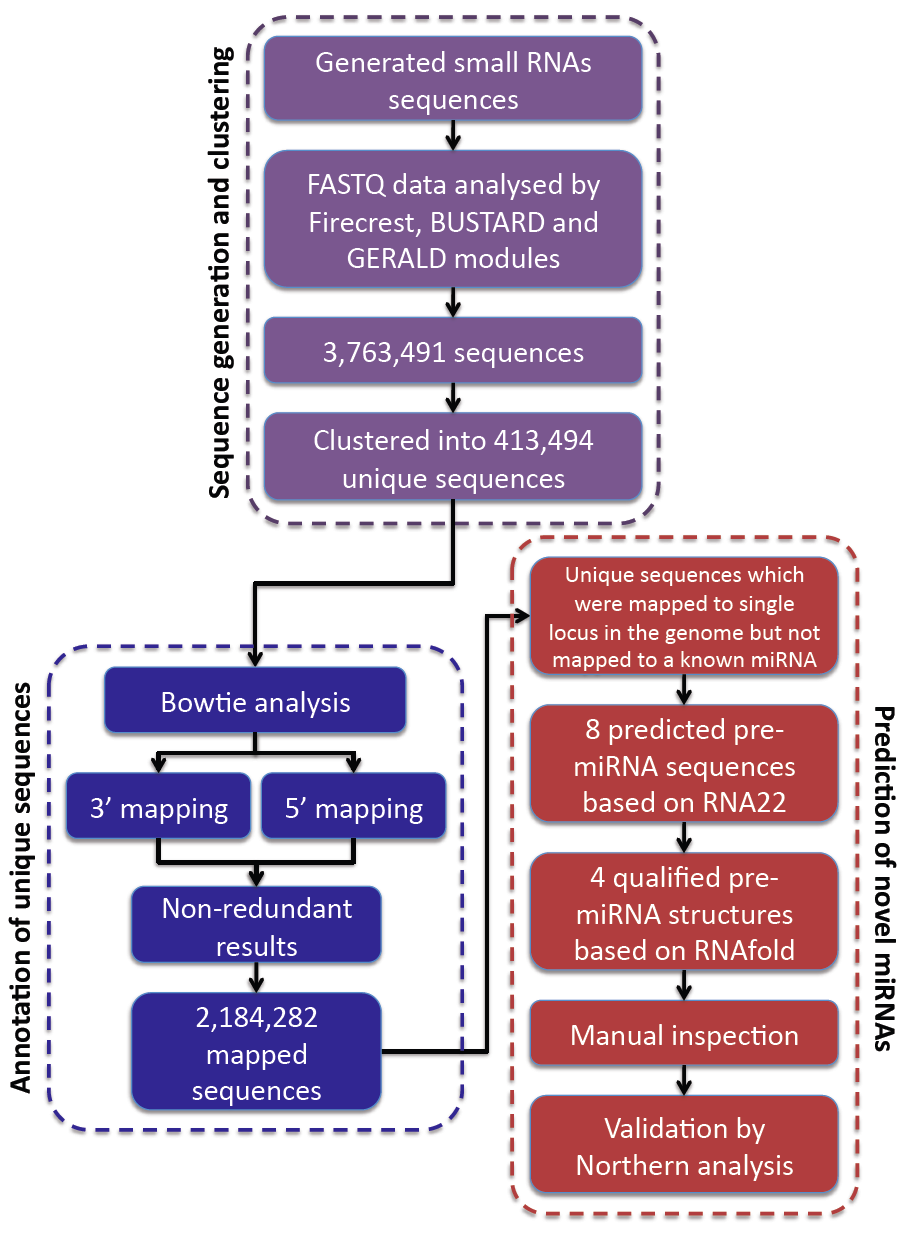


**Figure S1** Pipeline for sequence annotations and discovery of novel miRNA. The pipeline involves sequence generation and clustering followed by annotation of unique sequences and prediction of novel miRNAs.


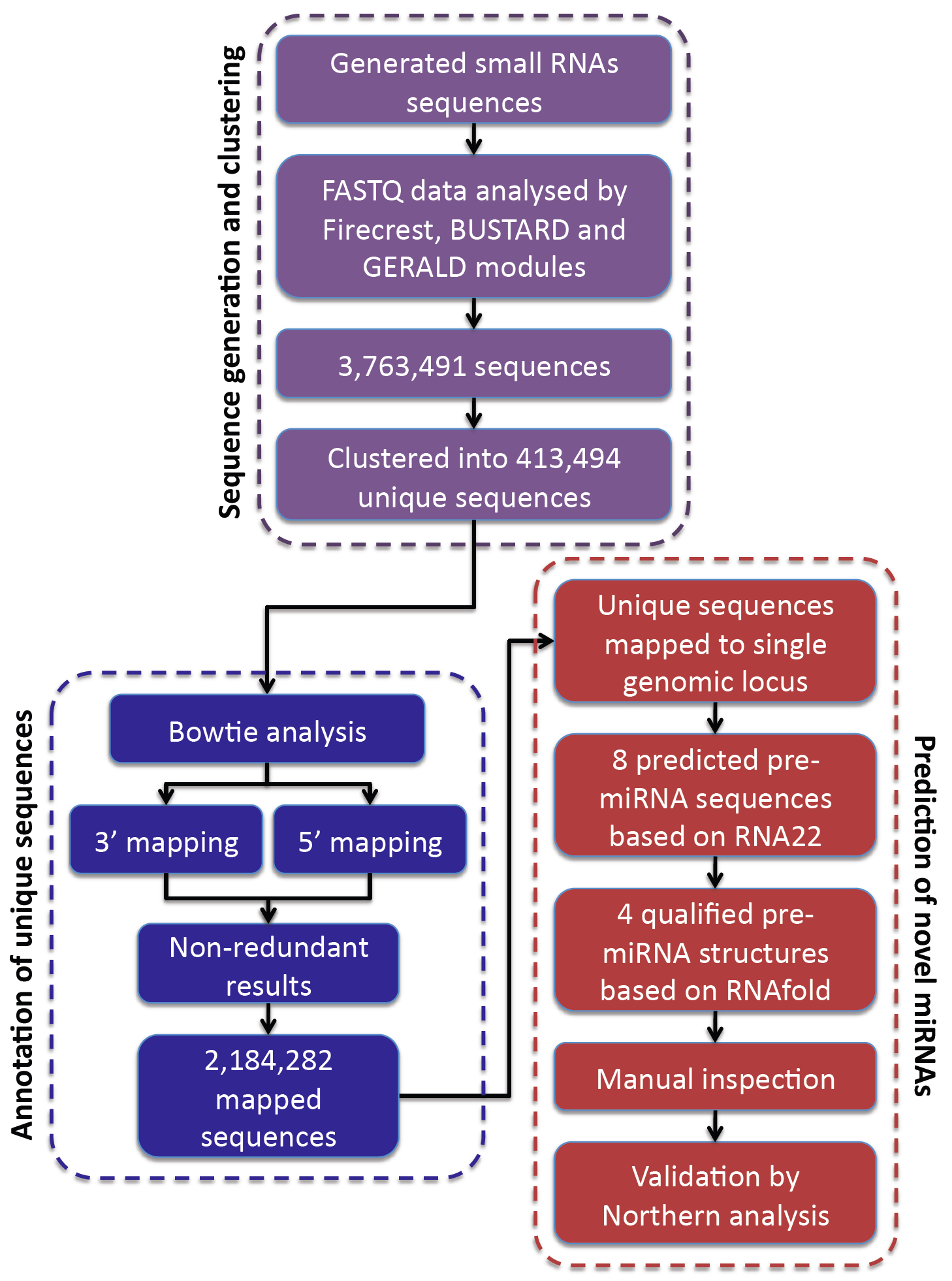


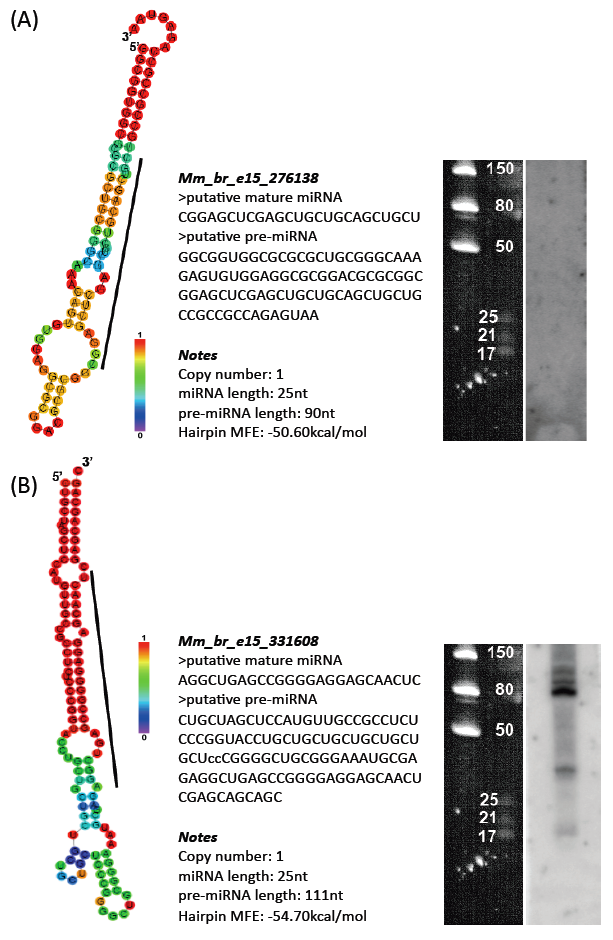


*The figure continues on next page…*

*
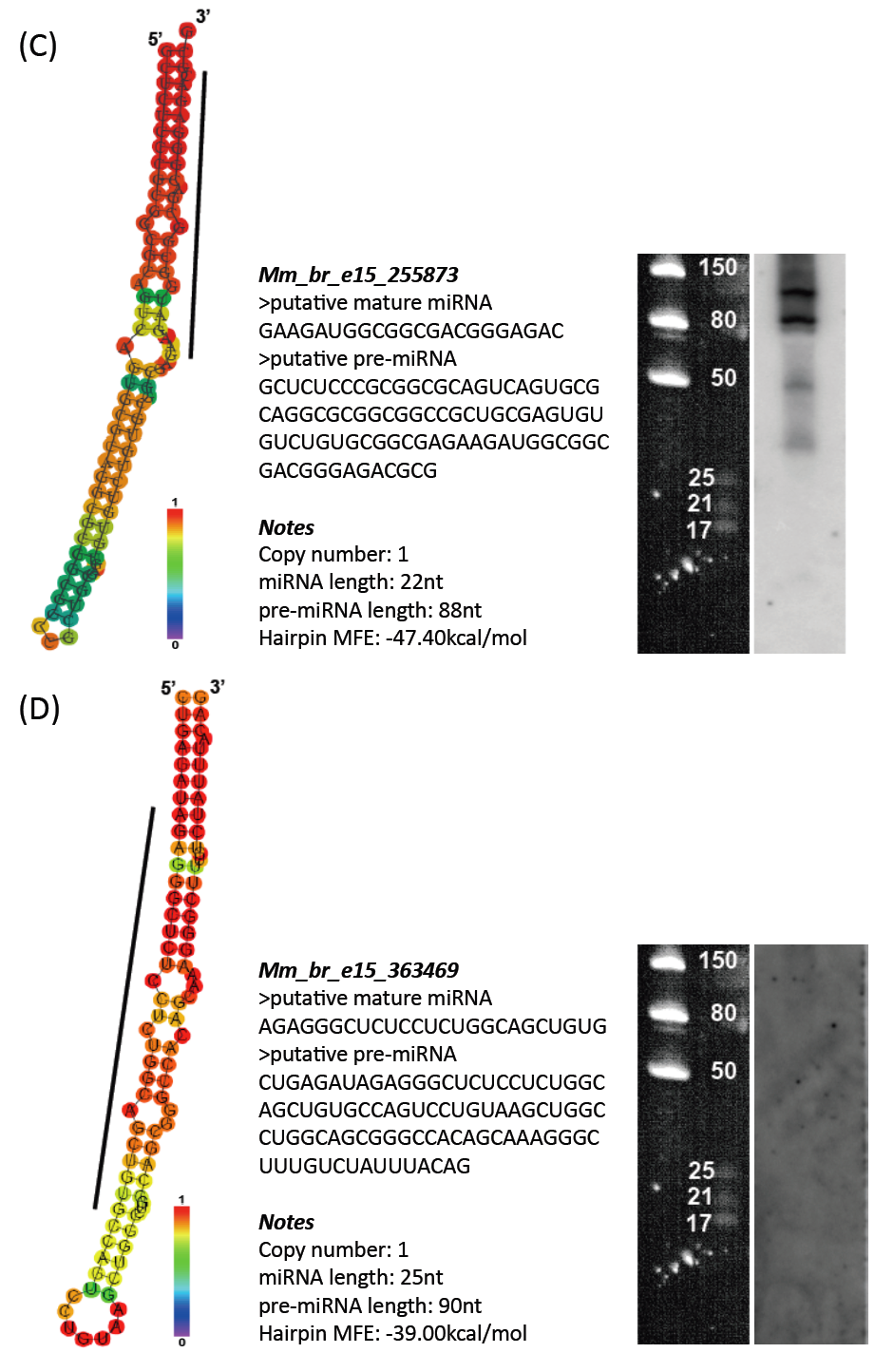
*

**Figure S2** Non-validated predicted pre- and mature miRNAs for mm_br_e15_276138 (A), mm_br_e15_331608 (B), mm_br_e15_255873 (C) and mm_br_e15_363469. These sequences were rejected due to either one or combination of the following features: a large internal loop (A), branching stemloops (B) or an oversize precursor structure (A-D). Northern blot analysis for all predicted pre- and mature miRNAs did not validate the existence of these putative miRNAs.

**Statistical information and analysis results of *miR-3099* expression**

**Experiment (A): Expression of *miR-3099* in mouse embryonic stem cells:**

***Purpose:*** To determine if DICER1 enzyme activity effects the expression levels of *miR-3099* in embryonic stem cells.

***Experimental Treatments:*** Embryonic stem cells with (wild type) and without (knock out) DICER1 enzyme activity.

***Statistical methods:*** One way Analysis of Variance.

***Results:*** Based on three replicates in each group, there was a significant difference in the expression levels in the stem cells with and without DICER1 activity (P<0.01). Wild type cells had a higher expression level (-2.86 ± 0.52) than the knock out cells (-8.16 ± 0.52).

**Experiment (B): Expression of *miR-3099* in embryogenesis:**

***Purpose:*** To determine if the expression levels of *miR-3099* vary throughout embryogenesis.

***Experimental Treatments:*** Embryonic day 3 blastocysts, and day 7 embryos.

***Statistical methods:*** One way Analysis of Variance.

***Results:*** Based on three replicates in each group, there was a significant difference in the expression levels in the blastocysts and day 7 embryos (P<0.001). The blastocysts had a higher expression level (-3.45 ± 0.12) than 7-day old embryos (-6.69 ± 0.12).

**Experiment (C): Expression of *miR-3099* in whole brains at different developmental stages:**

***Purpose:*** To determine if there were differences in the whole brain expression levels of *miR-3099* in the brain during development.

***Experimental Treatments:*** Whole brains of mice were taken at embryonic age 11.5, 13.5, 15.5 and 17.5 days, and postnatal days 1.5 and 150.

***Statistical Methods:*** One way Analysis of Variance.

***Results:*** There was a significant difference in the *miR-3099* expression levels in whole brains obtained from different ages (P=0.02). Whole brains at embryonic day 11.5 had significantly less expression than at any other age. There were no significant differences among the expression levels at the other ages. The LSDs are: 1) 1.31 for comparing postnatal Day 1.5 with any other age, and 1.44 for comparing any other ages. The means and standard errors for the whole brain expression at each age are given in the table below.

| Age | Number of replicates | Mean | SE |
| --- | --- | --- | --- |
| Embryonic Day 11.5 | 2 | -1.97 | 0.43 |
| Embryonic Day 13.5 | 2 | 0.30 | 0.43 |
| Embryonic Day 15.5 | 2 | -0.39 | 0.43 |
| Embryonic Day 17.5 | 2 | 0.27 | 0.43 |
| Postnatal Day 1.5 | 3 | 0.86 | 0.35 |
| Postnatal Day 150 | 2 | -0.12 | 0.43 |

**Experiment (D): Expression in different brain regions of mature mice:**

***Purpose:*** To determine if there are differences in expression levels of *miR-3099* among the different brain regions in adult mice.

***Experimental Treatments:*** The following brain regions were excised from adult mice (postanatal day 150): cerebellum; olfactory bulb; cerebrum; medulla; thalamus; and hippocampus.

***Statistical methods:*** One way Analysis of Variance.

***Results:*** There was no significant difference in the expression levels among the brain regions (P=0.45). The mean log2 normalized expression level is given in the following table for each brain region. For each region there are 2 replicates, and the standard error is 0.79.

| Brain region | Mean |
| --- | --- |
| Cerebellum | -3.28 |
| Olfactory bulb | -2.95 |
| Cerebrum | -2.56 |
| Medulla | -1.62 |
| Thalamus | -1.44 |
| Hippocampus | -1.38 |

**Experiment (E): Expression in different organs in adult mice:**

***Purpose:*** To determine if there are differences in expression levels of *miR-3099* among the different organs in adult mice.

***Experimental Treatments:*** The following organs were taken from adult mice (postnatal day 150): spleen; stomach; liver; skeletal muscle; skin; ovary; testes; brain; kidney; small intestine; heart; large intestine; thymus and pancreas.

***Statistical methods:*** One way Analysis of Variance.

***Results:*** There was a significant difference in expression levels among the organs (P<0.001). The mean log2 normalized expression level is given in the following table for each organ.

| Organ | Mean | Group |
| --- | --- | --- |
| Spleen | -4.88 | A |
| Stomach | -4.33 | A |
| Liver | -3.57 | B |
| Skeletal muscle | -2.69 | A |
| Skin | -1.77 | B |
| Ovary | -1.21 | B |
| Testes | -0.13 | B |
| Brain | -0.12 | B |
| Kidney | -0.01 | A |
| Small intestine | 0.32 | B |
| Heart | 1.48 | A |
| Large intestine | 1.98 | B |
| Thymus | 2.69 | B |
| Pancreas | 7.03 | B |

Group A organs have three replicates, and their standard error is 0.88, whereas group B organs have two replicates, and their standard error is 1.08. The least significant differences (LSDs) are: 2.61 when comparing two organs from group A; 3.19 when comparing two organs from group B; and 2.91 when comparing a group A organ with a group B organ.

**Experiment (F): Expression in different cell stages in P19 cells:**

***Purpose:*** To determine if there is a difference in expression levels of *miR-3099* between P19 neurodifferentiating and proliferating cells.

***Experimental Treatments:*** P19 cells were treated to differentiate of proliferate.

***Statistical methods:*** One way Analysis of Variance.

***Results:*** There was a significant difference in the expression levels in the different cell types (P=0.04). Differentiating cells had a higher expression level than the proliferating cells. The mean log2 normalized expression level is given in the following table for each cell stage.

| Cell Type | Number of replicates | MEAN | SE |
| --- | --- | --- | --- |
| Differentiating | 3 | -2.72 | 0.21 |
| Proliferating | 2 | -3.85 | 0.26 |
